# First Report of Sesame Mutants Tolerant to Severe Drought Stress during Germination and Early Seedling Growth Stages

**DOI:** 10.3390/plants10061166

**Published:** 2021-06-08

**Authors:** Mohamed Kouighat, Hafida Hanine, Mohamed El Fechtali, Abdelghani Nabloussi

**Affiliations:** 1 Research Unit of Plant Breeding and Plant Genetic Resources Conservation, National Institute of Agricultural Research, Regional Agricultural Research Center of Meknes, Meknes 50000, Morocco; mohammed.kouighat@usms.ac.ma (M.K.); mohamed.elfechtali@inra.ma (M.E.F.); 2 Laboratory of Bioprocess and Bio-Interfaces, Faculty of Science and Technology, University Moulay Slimane, Béni-Mellal 23000, Morocco; hafidahanine0@gmail.com

**Keywords:** abiotic stress, germplasm, oilseed crop, osmotic potential, *Sesamum indicum* L., stability

## Abstract

In the context of climate change and water scarcity, there is a need to develop and use drought-tolerant sesame cultivars. This study was conducted to evaluate the response of 13 sesame genotypes, including 11 mutants and their wild-types, to drought during germination and early seedling growth. Moderate and severe drought stress was simulated by applying polyethylene glycol (PEG) at two osmotic potentials, −0.6 MPa and −1.2 MPa, respectively, on seeds of two successive mutant generations, M_2_ and M_3_. The parameters measured or calculated were germination percentage (GP), germination rate (GR), mean germination time (MGT), root length (RL), shoot length (SL), root to shoot ratio (RSR), and the seedling vigor index (SVI). Results showed the significant effect of genotype, drought, and drought × genotype interaction on all parameters investigated. Under severe drought, seeds of seven genotypes, including wild types, were not able to germinate. There was a drastic decline of all parameters for the rest, except MGT and RSR, which markedly increased. Interestingly, two mutants, “ML2-5” and “ML2-10”, were identified as the most tolerant to severe drought and the most stable over both generations. The present work is the first report of sesame germplasm with such a high level of tolerance to drought during germination and early seedling growth stages.

## 1. Introduction

Sesame (*Sesamum indicum* L.) is known as “the queen of oilseeds” due to the high oil content and nutritional quality that characterize its seed [1]. Sesame seeds are rich in oil, protein, carbohydrates, vitamins, nutrients, antioxidants, and minerals as important nutritional sources for human health [2,3]. Sesame is a tropical and subtropical crop, but it is also cultivated under arid and semiarid climate conditions [4]. In 2019, the world’s cultivation area of sesame was around 12.82 Mha, ensuring a world production of about 6.55 Mt, of which about 60% comes from Asia [5].

Unlike other oilseed crops, sesame is reported to be more tolerant to drought [6]. However, in arid and semiarid areas, drought often occurs conjointly with heat or high temperatures and impairs sesame production significantly [2,3]. Harmful effects on sesame seed yield and quality are markedly noticed when this water stress happens, especially at germination and flowering stages [7,8,9,10]. Severe or prolonged drought adversely influences sesame productivity by reducing the number of capsules per plant, the yield, and the quality of the oil [11,12,13]. Drought stress can also affect the level of secondary metabolites and morphophysiological characteristics of the sesame seed [14]. Besides, drought becomes more damaging during flowering as it increases the susceptibility of the plant to attack from pathogens [15]. Seed germination is the first critical and most sensitive stage of the plant life cycle [6,16,17], because of its direct and strong correlation with the seedling establishment and early growth. Indeed, soil moisture is a determining factor in seed germination, and any reduction in osmotic potential associated with moisture decline delays or inhibits germination [18]. As a result of climate change, there is an increased drought frequency throughout the crop cycle, including at the germination stage. However, the magnitude of decrease in germination and early seedling growth depends upon the drought level and genotype [7,8,11,19,20]. Therefore, there is a need to improve crop tolerance to drought by breeding and selecting appropriate germplasm to be used for the development of adapted cultivars that could enable sustainable and competitive sesame cultivation in arid and semiarid environments. However, just a few studies have been carried out on sesame tolerance to drought during germination and early seedling growth stages by using and investigating different genotypes [8,11,12,19,20]. Besides, and to our knowledge, only one study has been performed on sesame mutants that were obtained through gamma-ray treatment [8].

In Morocco, drought has always been present as a climate structural element, with an increased frequency during the last decades due to climate change [21]. Furthermore, this Mediterranean country is characterized by low and irregular rainfall, often observed throughout all crop stages. In this context, sesame is cultivated on about 860 ha, ensuring an annual average production of 688 t [22]. This minor crop is mainly grown in the Tadla area as a catch crop, planted just after cereals are harvested in late spring, and harvested at the beginning of autumn. Therefore, this crop is fully irrigated to overcome drought and high evaporative demand occurring during the plant life cycle, leading to salinization of the soil and, thus, to deterioration of its quality, besides the increasing waste of water. Therefore, there is a need to grow drought-tolerant cultivars to reduce irrigation frequency from the germination stage until plant maturity. As a result, a large amount of irrigation water will be saved, and the soil will remain healthier. This would be a relevant and sound strategy to promote and develop this crop in Morocco and other African areas. 

The cultivars currently available in Morocco are genetically very close, as shown by recent studies based on phenotypical traits [23] and molecular markers [24]. Thus, a mutagenesis breeding program was launched to broaden the existing genetic variability, using the chemical mutagen ethyl methane sulfonate (EMS). As a result, several interesting mutants were induced, exhibiting superiority when compared to their wild-types as regards some morphological and agronomic traits [25]. It would be noteworthy to investigate the reaction of these mutants to early drought happening at the germination stage. To estimate the effect of drought stress on seed germination, solutions with variable osmotic potentials are effective [26]. Osmotic stress at the germination stage is often simulated by chemical molecules such as polyethylene glycol (PEG), mannitol, sodium chloride (NaCl), sucrose, and glucose [8,11]. PEG is one of the molecules most commonly used for this purpose [27]. A previous study on Moroccan cultivars showed that yellow and brown seeds germinated better than white and black seeds in the presence of drought stress (PEG) at germination [20]. Germination was more reduced and delayed as the drought level increased, i.e., lowering the osmotic potential until −1.2 MPa of PEG, for which no germination was recorded. Therefore, the objective of the present study is firstly to assess the response of some sesame mutants (generations M_2_ and M_3_) to moderate (−0.6 MPa) and severe drought stress (−1.2 MPa of PEG-6000), at germination and early seedling growth, and secondly to select the most drought-tolerant mutants.

## 2. Results

### 2.1. Drought Stress Effects on Germination

**Figure 1:**
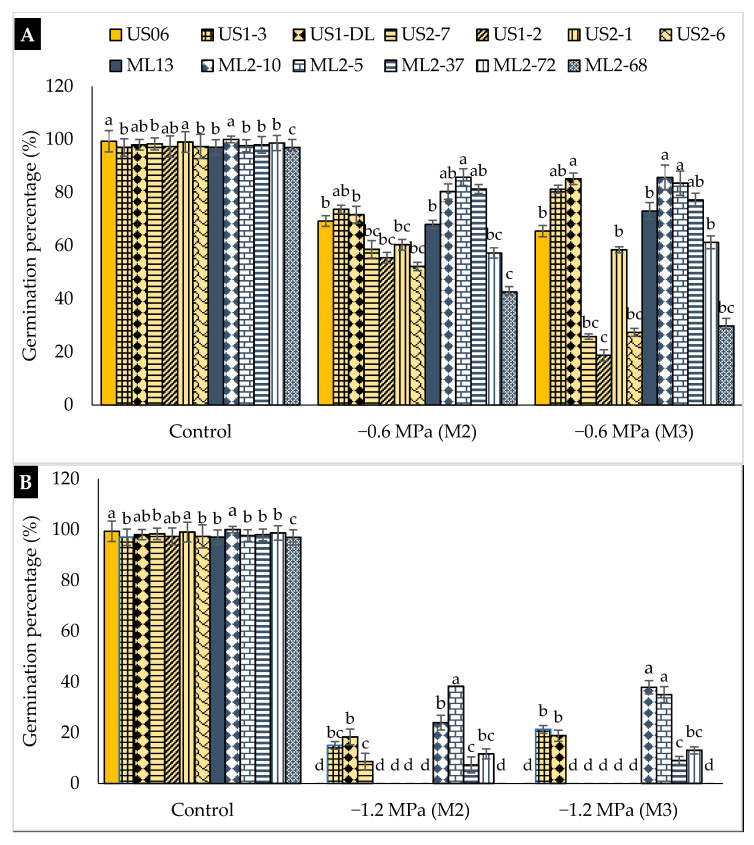
The effect of drought stress on germination percentage (GP) in 11 sesame mutants and their two wild types evaluated over two generations, M_2_ and M_3_: (**A**) moderate stress (−0.6 MPa); (**B**) severe stress (−1.2 MPa). Values with different alphabetical superscripts are significantly different (*p* ≤ 0.05) according to Duncan’s new multiple range test (DMRT).

The analysis of variance results, summarized in Table 1, showed that drought significantly affected all germination parameters tested in M_2_ and M_3_ generations. Significant variation among both generations was recorded only for germination percentage (GP), while for germination rate (GR) and mean germination time (MGT) no significant difference was observed. Similarly the interaction genotype × generation was significant only for GP, indicating the stability of genotypes for GR and MGT through both generations. On the other hand, there was a significant effect (*p* < 0.05) of genotype and genotype × drought interaction on GP, GR, and MGT (Table 1). As shown in Figure 1, the highest values of GP were observed in the absence of stress (control), ranging from 97.00 (“ML2-68”) to 100% (“ML2-10”). This parameter was significantly reduced in all genotypes exposed to PEG. At moderate stress (−0.6 MPa), GP was significantly reduced in “ML2-68” (42%) followed by “US2-6” (52%) and “US1-2” (55%) in the M_2_ generation, and “US1-2” (18%) followed by “US2-7” (25%) and “US2-6” (27%) in the M_3_ generation (Figure 1A). On the other hand, “ML2-5” showed the highest GP, 85.78%, followed by “ML2-37” (81.32%) and “ML2-10” (80.36%) in the M_2_ generation, while “ML2-10”, “US1-DL”, and “ML2-5” exhibited the highest GP values in M_3_, namely 85.69, 85.17, and 83.55% respectively. Under severe drought (−1.2 MPa), seed germination was completely inhibited in genotypes “US06”, “US2-6”, “US2-1”, “US1-2”, “ML13”, and “ML2-68” (Figure 1B). For the rest of the genotypes, and in the M_2_ generation, the highest values of GP were 38.33%, recorded in “ML2-5”, and 24% observed in “ML2-10”. In the M_3_ generation, the mutant “ML2-10” was the most tolerant, having a GP of 37.91%, followed by the mutant “ML2-5”, with 35.02%. This indicates the stability of these two mutants for drought tolerance during germination. Nevertheless, no germination was recorded for “US2-7” in the M_3_ generation, while it showed a GP of 8.66% in the M_2_ generation. This might indicate this mutant has lost the potential to tolerate severe drought during seed germination from the M_2_ to M_3_ generation.

**Figure 2:**
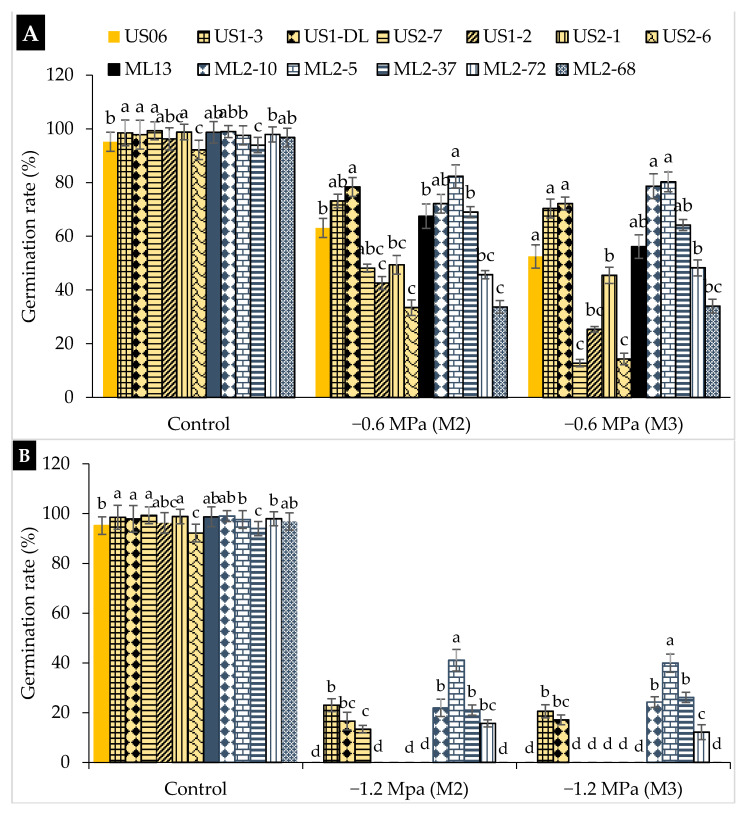
The effect of drought stress on germination rate (GR) in 11 sesame mutants and their two wild types evaluated over two generations, M_2_ and M_3_: (**A**) moderate stress (−0.6 MPa); (**B**) severe stress (−1.2 MPa). Values with different alphabetical superscripts are significantly different (*p* ≤ 0.05) according to DMRT.

Seed germination rate (GR) was significantly (*p* < 0.05) higher in control seeds (0 MPa PEG) than treated ones (subject to stress), with a variation from 92.15 to 99.27% in “US2-6” and “US2-7”, respectively. For moderate stress (−0.6 MPa), the mutants “ML2-5”, “US1-DL”, “US1-3”, and “ML2-10” showed the highest GR values in the M_2_ generation, namely 82.30, 78.36, 73.14, and 72.14%, respectively (Figure 2A). Similarly, in the M_3_ generation, the germination rate was higher in “ML2-5” (80%), “ML2-10” (78%), “US1-DL” (72%), and “US1-3” (70%), compared to the other genotypes. On the other hand, low GR values were recorded for “US2-6” (33%), “ML2-68” (33%), and “US1-2” (42%) in the M_2_ generation, and for “US2-7” (12%), “US2-6” (14%), and “US1-2” (25%) in the M_3_ generation (Figure 2A). At severe drought stress (−1.2 MPa), GR decreased significantly (*p* < 0.05) in all genotypes tested (Figure 2B). Once again, “ML2-5” confirmed its tolerance to severe drought stress, maintaining the highest GR (40%) compared to other genotypes. In the M_2_ generation, it had a GR of about 41.10%, followed by “US1-3” (23%) and “ML2-10” (21%), and in the M_3_ generation, it had 40%, followed by “ML2-37” (26%) and “ML2-10” (24%) (Figure 2B). This indicates the stability of “ML2-5” for GR through both M_2_ and M_3_ generations. 

**Figure 3:**
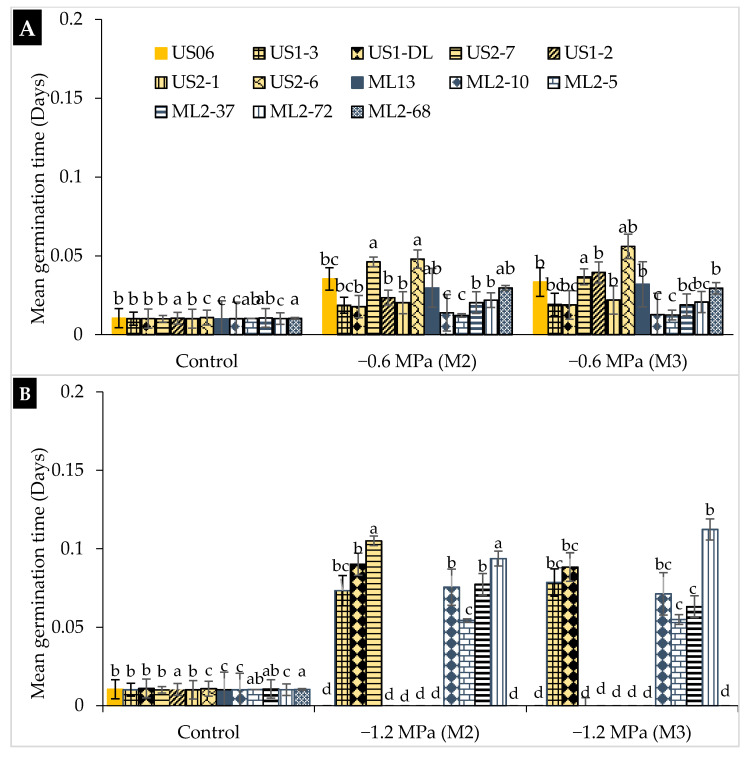
The effect of drought stress on mean germination time (MGT) in 11 sesame mutants and their two wild types evaluated over two generations, M_2_ and M_3_: (**A**) moderate stress (−0.6 MPa); (**B**) severe stress (−1.2 MPa). Values with different alphabetical superscripts are significantly different (*p* ≤ 0.05) according to DMRT.

For mean germination time (MGT), which tells us how fast seeds germinate, we observed that there were significant differences (*p* < 0.05) among genotypes under water stress conditions. Overall, MGT increases with water stress (Figure 3). It was about 0.01 day (d) for all genotypes in the absence of drought stress and much longer under moderate and severe stress conditions. In fact, MGT ranged from 0.012 d in “ML2-5” to 0.051 d in “US2-6” under moderate stress, and from 0.05 d in “ML2-5” to 0.11 d in “ML2-72” under severe stress. For moderate drought, the mutants “ML2-5” and “ML2-10” showed the lowest MGT values, namely 0.012 and 0.013 d, respectively, both in M_2_ and M_3_ generations (Figure 3A). Regarding severe stress, and in the M_2_ generation, MGT was 0.054, 0.073, and 0.075 d in “ML2-5”, “US1-3”, and “ML2-10”, respectively, whilst in the M_3_ generation, it was 0.055, 0.063, and 0.071 d in “ML2-5”, “ML2-37”, and “ML2-10”, respectively (Figure 3B). The two mutant genotypes, “ML2-5” and “ML2-10”, showed fast germination even under severe drought conditions, for both M_2_ and M_3_ generations, which indicates their stable genetic ability to tolerate a high level of drought. 

### 2.2. Drought Stress Effects on Early Seedling

ANOVA results showed, in both generations, a significant effect (*p* < 0.05) of drought, genotype, and drought × genotype interaction on all early seedling growth parameters, namely shoot length (SL), root length (RL), root-to-shoot ratio (RSR), and the seedling vigor index (SVI) (Table 1). However, among both generations, significant variation was only recorded for RSR and the SVI, suggesting there was a stability of the genotypes studied for SL and RL over M_2_ and M_3_ generations.

**Figure 4:**
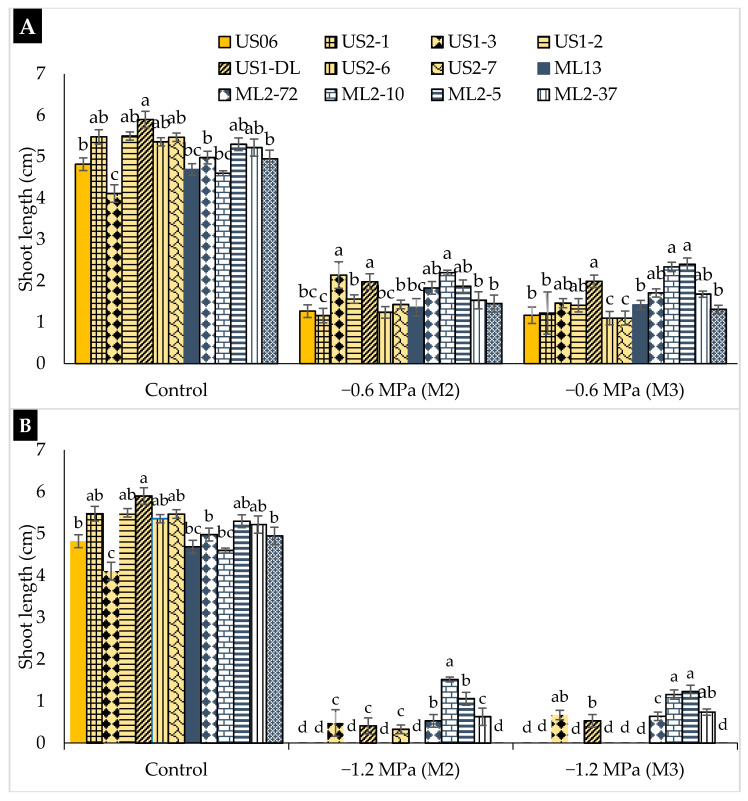
The effect of drought stress on shoot length (SL) in 11 sesame mutants and their two wild types evaluated over two generations, M_2_ and M_3_: (**A**) moderate stress (−0.6 MPa); (**B**) severe stress (−1.2 MPa). Values with different alphabetical superscripts are significantly different (*p* ≤ 0.05) according to DMRT.

Figure 4 presents the mean values of shoot length (SL) in the presence and absence of water deficit. In the absence of stress (control), the highest mean SLs of 5.90, 5.50, and 5.48 cm were observed in “US1-DL”, “US1-2”, and “US2-1”, respectively. In the presence of drought, SL decreased in all genotypes. At a moderate water potential (−0.6 MPa), the most remarkable shoot length (about 2 cm) was observed in “ML2-10” and “US1-3”, while the shortest SL (about 1 cm) was found in “US1-DL”, “US2-6”, and “US2-7” in both generations (Figure 4A). In the case of severe water stress (−1.2 MPa), the two mutants “ML2-10” and “ML2-5” confirmed their early growth potential and stability under stressful conditions. Shoots of “ML2-10” reached 1.52 and 1.16 cm length in generations M_2_ and M_3_, respectively, while those of “ML2-5” outreached 1.06 and 1.23 cm, respectively (Figure 4B). 

**Figure 5:**
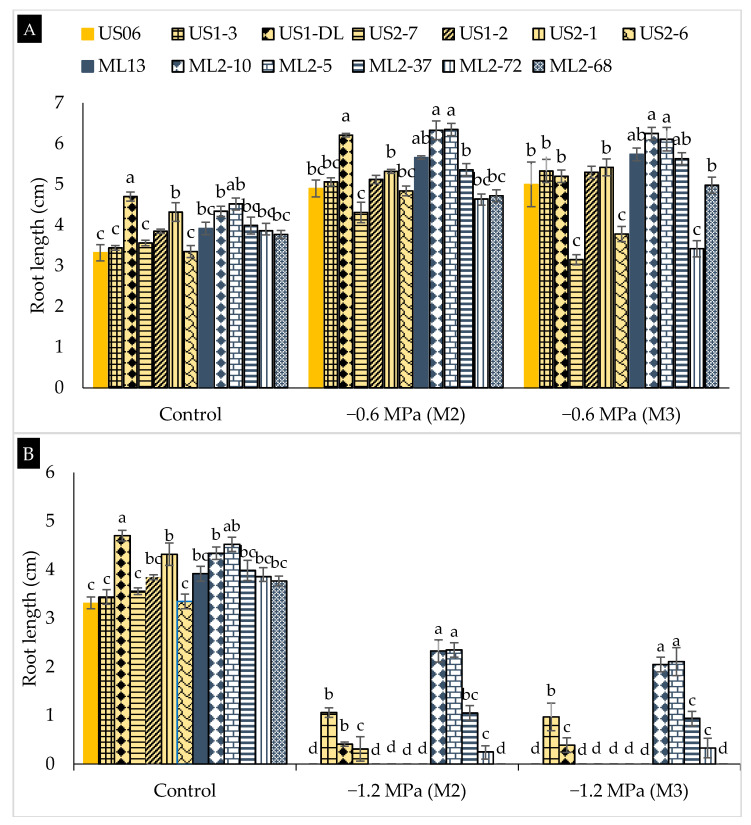
The effect of drought stress on root length (RL) in 11 sesame mutants and their two wild types evaluated over two generations, M_2_ and M_3_: (**A**) moderate stress (−0.6 MPa); (**B**) severe stress (−1.2 MPa). Values with different alphabetical superscripts are significantly different (*p* ≤ 0.05) according to DMRT.

Regarding root length (RL), and in the absence of any water stress, the genotypes “US1-DL” and “ML2-5” exhibited the highest average values, 4.70 and 4.52 cm, respectively. In contrast, “US06” and “US2-6” recorded the lowest average RLs, 3.32 and 3.35 cm, respectively (Figure 5). Overall, RL increased under moderate drought conditions in all genotypes and for both seed generations, particularly in “ML2-10” and “ML2-5”, which maintained their superiority (more than 6 cm) in both M_2_ and M_3_ generations (Figure 5A). For severe water stress, a strong decrease in RL was noted in all genotypes (Figure 5B). Again, and unlike the other genotypes, “ML2-5” and “ML2-10” showed and retained the lowest reduction in RL of 47 and 46%, respectively, in the M_2_ generation, and 53 and 52%, respectively, in the M_3_ generation. This indicates that “ML2-10” and “ML2-5” are efficient and stable in developing high root system growth even in the presence of severe drought. 

**Figure 6:**
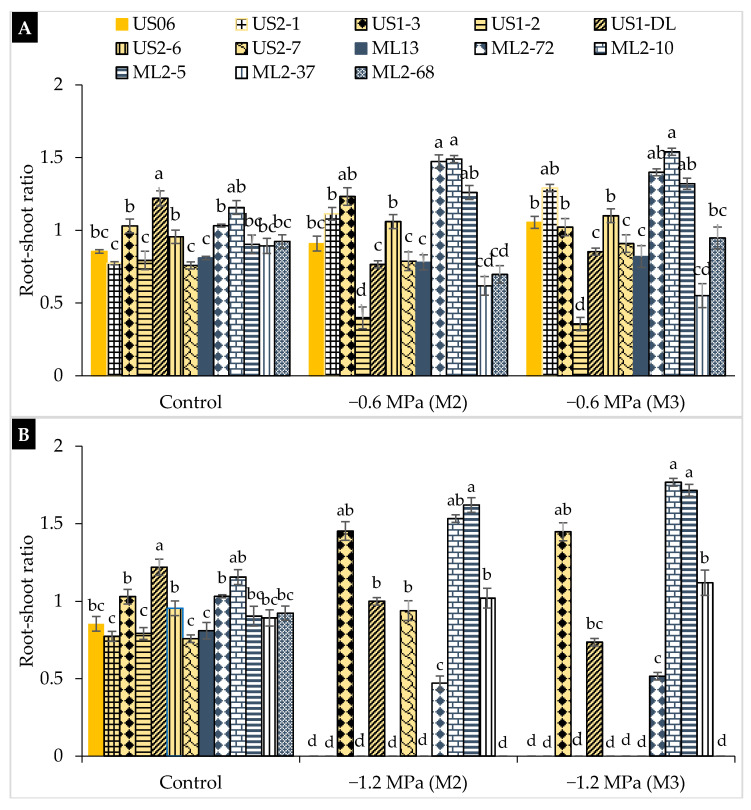
The effect of drought stress on root to shoot ratio (RSR) in 11 sesame mutants and their two wild types evaluated over two generations, M_2_ and M_3_: (**A**) moderate stress (−0.6 MPa); (**B**) severe stress (−1.2 MPa). Values with different alphabetical superscripts are significantly different (*p* ≤ 0.05) according to DMRT.

**Figure 7:**
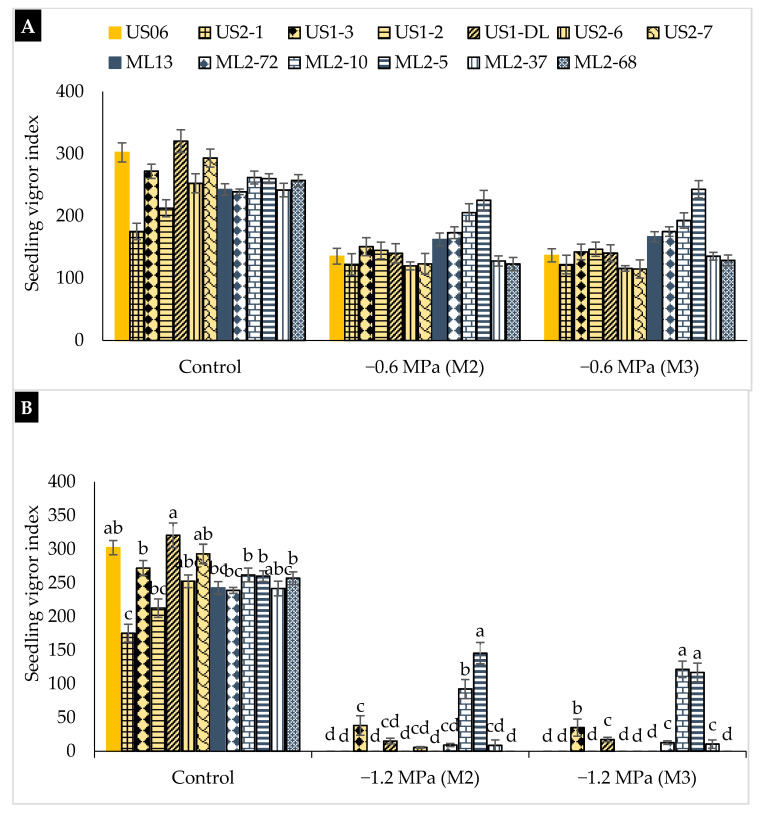
The effect of drought stress on the seedling vigor index (SVI) in 11 sesame mutants and their two wild types evaluated over two generations, M_2_ and M_3_: (**A**) moderate stress (−0.6 MPa); (**B**) severe stress (−1.2 MPa). Values with different alphabetical superscripts are significantly different (*p* ≤ 0.05) according to DMRT.

Under non-stressful conditions, the root to shoot ratio (RSR) ranged from 0.75 cm in “US2-7” to 1.22 cm in “US1-DL”. Except for “US1-2”, “ML2-37”, and “US1-DL”, all the genotypes studied showed an increasing trend in RSR by reducing the osmotic potential from 0 to −1.2 MPa (Figure 6). At moderate stress (−0.6 MPa), the RSR ranged from 0.39 (“US1-2”) to 1.49 (“ML2-10”) in the M_2_ generation, and from 0.35 (“US1-2”) to 1.54 (“ML2-10”) in the M_3_ generation (Figure 6A). At severe stress (−1.2 MPa), the highest RSR values were recorded in “ML2-5” (1.61 in M_2_ and 1.71 in M_3_) and “ML2-10” (1.53 in M_2_ and 1.76 in M_3_) (Figure 6B). An increase in RSR indicates that a seedling is growing under unfavorable conditions. Once again, the mutants “ML2-10” and “ML2-5” confirm their performance and stability by exhibiting the highest RSR under both moderate and severe drought stresses. Regarding the seedling vigor index (SVI), the highest values were found under non-stress conditions, with a variation from 175.36 in “US2-1” to 320.63 in “US1-DL” (Figure 7). Under moderate water stress (−0.6 MPa), the SVI decreased significantly, ranging from 119.89 (“US2-6”) to 225.48 (“ML2-5”) in the M_2_ generation and from 114.96 (“US2-7”) to 243.20 (“ML2-5”) in the M_3_ generation (Figure 7A). Under severe drought (−1.2 MPa), the mutants, “ML2-5” and “ML2-10” showed the highest SVI values, namely 145.67 and 116.97 in M_2_ and 92.40 and 121.69 in M_3_, respectively (Figure 7B), which suggests and confirms their tolerance to severe drought occurring at early seedling growth. 

Finally, average values of all studied parameters, regarding both seed germination and early seedling growth, are shown in the supplementary material Table S1.

## 3. Discussion

### 3.1. Drought Stress Effects on Germination

In the present study, all measured seed germination parameters, GP, GR, and MGT, were affected by drought in all genotypes studied, with a decrease in GP and GR and an increase in MGT, as a response to stress exposure. This is in agreement with findings of previous studies in sesame [8,12,19,20], as well as in other oilseed crops like rapeseed [28,29], sunflower [30], and safflower [21]. This may be due to the alteration of enzymes and hormones present in the seed or the generation of free radicals, which alter the metabolic pathways in seeds germinating under drought stress [31,32]. As a result of pronounced seed dehydration, there is an alteration of mechanisms leading to embryo development [33]. In fact, seed germination is directly linked to the use of reserves, respiration, and phytohormones that are all affected during the development of the embryo in the stressed seed [34,35]. Therefore, the development of genotypes with higher seed metabolic efficiency under drought conditions is a desirable crop improvement trait. In our case, all genotypes were slightly affected by moderate water stress. However, under severe drought conditions, their seed germination was drastically reduced. In some of these genotypes, germination was inhibited in both generations, M_2_ and M_3_. These results are in agreement with those of Boureima et al. [8], Harfi et al. [20], and Dissanayake et al. [12], who reported that the germination of sesame seeds is completely inhibited by a water potential lower than –1 MPa of PEG. However, some mutants kept germinating in this particular situation, showing their tolerance to such a high moisture stress level. Among them, the mutants “ML2-5” and “ML2-10” are the most interesting as they were stable over both generations M_2_ and M_3_, and the least affected, with a respective average reduction of less than 63 and 70%. This is, so far, the first report of sesame genetic materials that are able to germinate at an osmotic potential of −1.2 MPa. Germination rate (GR) is one of the indices used to evaluate crop tolerance to drought stress. A reduction in germination rate and total germination is one of the most common responses of drought-exposed plants [36,37]. Our results showed that GR decreases significantly with water deficit, which is in agreement with the results of El Harfi et al. [20] and Bakhshandeh et al. [38] in sesame. In the present study, the mutants “ML2-5” and “ML2-10” had the highest GR values, over both generations, M_2_ and M_3_, under moderate (80% and 75%, respectively) and severe (40% and 25%, respectively) moisture stress.. The mutant “ML2-5” is of particular interest as it exhibited not only the highest germination rate but also the lowest mean germination time (MGT) under severe drought conditions. It is well known that in such conditions, the seed requires more time to adjust its internal osmotic potential to the external environment [39] and, thus, takes more time to germinate compared to non-stressful conditions. However, a delay in the MGT can be harmful to the successful establishment of a crop stand. Therefore, and based on all seed germination parameters studied, the mutant “ML2-5”, followed by the mutant “ML2-10”, is the most tolerant to high levels of moisture stress. They both could be considered as novel and relevant drought-tolerant germplasms, during the germination stage, since no similar sesame material has been found and reported in the literature.

### 3.2. Drought Stress Effects on Early Seedling

A remarkable diminution in shoot length was observed with a decrease in water potential from 0 to −1.2 MPa. This may be due to the high sensitivity of shoot tissues to water deficit [40]. Similar results were reported in sesame by Mensah et al. [19], El Harfi et al. [20], and Dissanayake et al. [12]. Our findings show that “ML2-10” and “ML2-5” are the most drought-tolerant among the genotypes studied. In fact, under moderate stress, “ML2-10” and “ML2-5” developed SLs of about 2 cm in both M_2_ and M_3_ generations, and even under severe stress these two mutant lines maintained SLs above 1 cm over the two generations. In previous studies on sesame, no shoot growth was reported for similar severe drought conditions. Additionally, root length was drastically reduced by severe water stress (−1.2 MPa). However, it was improved under moderate stress, compared to non-stress conditions (control). The decrease in root length could be due to reduced cell multiplication in root meristems [41]. Similar findings were reported by Boureima et al. [8], who described that root length increased under moderate stress (−0.5 MPa) and decreased at severe stress (−1 MPa) in Senegalese sesame. Likewise, other previous studies have shown a significant reduction in root length under severe stress [12,19,20,42]. In our research, the longest roots were found in “ML2-10” and “ML2-5”, both under severe and moderate stress and over both generations. These findings are interesting in terms of early drought-tolerance and genetic stability. Admittedly, root traits are the first to be affected under drought stress conditions, and genotypes exhibiting better performance may be more tolerant [43]. Additionally, a plant’s ability to develop an extensive root system contributes to its drought tolerance [44]. Thus, root morphology and/or growth rate may be promising markers for selecting drought-tolerant varieties [45,46].

The root-to-shoot ratio (RSR) reflects the manner in which roots develop with regard to the growth of a plant. A high RSR indicated the faster growth of the root compared to the shoot. Our results showed that moderate and severe stresses led to the increase of this ratio in most of genotypes. The RSR for the genotypes tested varied considerably, “ML2-5”, “US1-3”, and “ML2-10” were the most tolerant to moderate and severe drought through both M_2_ and M_3_ generations, as they exhibited the highest RSR values. Finally, the seedling vigor index (SVI), combining germination and shoot growth, is more sensitive to drought and could be considered an effective indicator of drought tolerance in crops [47]. Our findings showed a diminution in the SVI after seedling’s exposure to drought, which is in agreement with reports of Spielmeyer et al. [48] in wheat and Koskosidis et al. [49] in chickpeas. The mutant lines “ML2-5” and “ML2-10” are the least affected, maintaining the highest SVI under both moderate and severe stresses and confirming, thus, their highest tolerance to moisture stress. 

Finally, significant differences were recorded between the two generations (M_2_ and M_3_) for GP, RSR, and the SVI, particularly in the mutants “US2-7”, “US1-2”, and “US1-3”. This could be explained by genetic factors related to these genotypes and pointed to the instability of the mutated genes related to these parameters. Contrarily, in most of the mutants studied, one could observe slight differences among both generations for the majority of the parameters investigated. This is particularly true for the most drought-tolerant mutants, “ML2-10” and “ML2-5”, which suggests their genetic stability and confirms their suitability to be used as relevant and appropriate germplasm in breeding programs aimed at improving drought tolerance in sesame during germination and early seedling growth stages.

## 4. Materials and Methods

### 4.1. Plant Material

This study’s plant material consists of 11 sesame mutant lines, along with their two wild-types (“ML13” and “US06”). Using chemical EMS-mutagenesis, these mutants were recently developed and characterized as described by Kouighat et al. [25]. They were selected based on some phenological, morphological, and agronomic traits. Their most important characteristics are summarized in Table 2. In the present work, seeds from both M_2_ and M_3_ generations were studied to confirm the results obtained and to assess the stability of the characters and behaviors observed in such mutant lines.

### 4.2. Seed Treatment under PEG-6000 Solution 

The experiment was carried out in 2020 at the oilseed crops laboratory in the Regional Center of Agricultural Research in Meknes, belonging to the “Institut National de la Recherche Agronomique” (INRA-Morocco). It was conducted in a completely randomized design, with two factors and three replications. The first factor was the genotype (mutants and parents), with 13 levels, and the second one was the drought stress, induced by the PEG solutions, with three levels of water potential, 0, −0.6, and −1.2 MPa, applied according to the equation of Michel & Kaufmann [50]. These potential levels were designed to simulate absence of water stress, moderate stress, and severe stresses, respectively. The choice of water potentials was based on previous studies showing that the use of the osmotic potential −1.2 MPa completely inhibited the germination of sesame seeds [8,20,21]. The seeds used in this study, from both M_2_ and M_3_ generations, were harvested in 2019 and 2020, respectively. For each genotype, 50 sesame seeds were immersed in 5% sodium hypochlorite for 5 min and then rinsed with distilled water to sterilize their surfaces. The seeds were incubated for germination on Whatman paper in glass Petri dishes (10 cm × 15 mm) at 24 ± 1 °C in the incubator. Every 48 h, 3 mL of PEG-6000 solution were added to the Petri dishes. However, in control (0 MPa), 3 mL of distilled water were added instead. The seeds were considered to be germinated when the radicle reached up 2 mm length [51].

### 4.3. Parameters Calculated/Measured and Statistical Analyses

The germinated seeds were counted at regular intervals every 48 h until the end of the experiment (eight days). The germination percentage (GP), calculated on day eight, as well as the germination rate (GR), were determined as follows: 

GP = (N8/50) × 100, and GR = Σ [(Gi − Gi − 1)/i], where N8 is the number of seeds germinated on the 8th day, Gi is the number of seed germinated on the day i, and Gi-1 is the number of seeds germinated on day i − 1 [52]. 

The mean germination time (MGT) is calculated according to the formula MGT = 1/GR. Root length (RL) and shoot (SL) length (cm), root to shoot ratio (RSR), and the seedling vigor index (SVI = (seedling length (cm) × germination percentage)/100) were determined according to Sadeghi et al. [53]. 

To determine statistically significant differences between genotypes, drought, and their levels of interaction, data were subjected to analysis of variance (ANOVA) with two factors, all considered as fix. Duncan’s new multiple range test (DMRT) was applied to compare treatment options and classify genotypes according to their tolerance to drought. These analyses were performed using the software package SPSS for Windows (version 20).

## 5. Conclusions

In conclusion, this is the first report of sesame mutants which are highly drought-tolerant and stable during seed germination and early seedling growth, managing to germinate and grow under severe drought corresponding to a water osmotic potential of −1.2 MPa. Nevertheless, an experiment under field conditions, with an irrigation deficit regime, would be useful to confirm such findings. The two mutants, “ML2-5” and “ML2-10”, could be managed and used as promising germplasms for developing cultivars with a high potential of germination under limited water availability conditions. Additionally, it would be interesting to assess the tolerance of these genotypes to drought stress occurring at different adult plant stages, particularly during flowering and grain-filling periods.

## Figures and Tables

**Table 1:** Analysis of variance (mean squares) for seed germination and seedling growth-related traits of 13 sesame genotypes evaluated under drought stress for two seed generations.

Source of Variation	Degree of Freedom	Germination Percentage	Germination Rate	Mean Germination Time	Seedling Vigor Index	Root Length	Shoot Length	Root/Shoot Ratio
Genotype (G)	12	418.43 ***	290.89 ***	0.070 ***	23,273.49 ***	3.12 ***	2.91 ***	18,970.66 ***
Drought (D)	2	147,508.51 ***	14,839.06 ***	0.0210 ***	919,444.07 ***	64.28 ***	354.17 **	3394.67 ***
Generation (M)	1	51.92 *	209.72	0.0117	490,553.31 **	0.02	3.12	413.98 *
G × D	24	139.17 ***	247.95 **	0.0060 ***	13,028.48 ***	1.29 ^***^	1.88 ***	9812.85 ***
M × G	12	403.02 *	221.93	0.067	25,990.86	3.05	2.63 *	19,039.58

*, **, *** Significant at 0.05; 0.01 and 0.001 probability levels, respectively.

**Table 2:** The main phenotypic characteristics of the studied sesame mutant lines.

Genotypes	Characteristics
“US06”	Parent (wild-type), white seeds, and one capsule/axil
“US2-7”	Mutant, white seeds, and high 1000-seed weight
“US1-DL”	Mutant, white seeds, and late maturity
“US1-3”	Mutant, white seeds, and early flowering
“US2-1”	Mutant, white seeds, and three capsules/axil
“US1-2”	Mutant, very large leaf, and high seed yield
“US2-6”	Mutant, pale black seeds, early flowering, and maturity
“ML13”	Parent (wild-type), beige seeds, and one capsule/axil
“ML2-5”	Mutant, brown seeds, and large capsule
“ML2-10”	Mutant, brown seeds, and high plant branching
“ML2-72”	Mutant, light brown seeds, and high 1000-seed weight
“ML2-37”	Mutant, beige seeds, and thick leaf
“ML2-68”	Mutant, grey seeds, and three capsules/axil

## Supplementary Materials

The following are available online at https://www.mdpi.com/article/10.3390/plants10061166/s1, Table S1: The average values of all parameters, the germination percentage (GP), the germination rate (GR), mean germination time (MGT), root length (RL), shoot length (SL), root-to-shoot ratio (RSR), and seedling vigor index (SVI) in both generations M_2_ and M_3_.

## References

[1] Moazzami A., Kamal-Eldin A. (2009). Sesame Seed Oil. Gourmet and Health-Promoting Specialty Oils.

[2] Anilakumar K.R., Pal A., Khanum F., Bawa A.S. (2010). Nutritional, Medicinal and Industrial Uses of Sesame (*Sesamum indicum* L.) Seeds-an Overview. Agric. Conspec. Sci..

[3] Hama J.R. (2017). Comparison of Fatty Acid Profile Changes between Unroasted and Roasted Brown Sesame (*Sesamum indicum* L.) Seeds Oil. Int. J. Food Prop..

[4] Arslan H., Ekin Z., Hatipoglu H. (2018). Performances of sesame genotypes (*Sesamum indicum* L.) with different seed shell colors in semi-arid climate conditions. Fresen. Environ. Bull..

[5] FAOSTAT. http://www.fao.org/faostat/fr/#data/QC.

[6] Islam F., Gill R.A., Ali B., Farooq M.A., Xu L., Najeeb U., Zhou W. (2016). Chapter 6—Sesame. Breeding Oilseed Crops for Sustainable Production.

[7] Hassanzadeh M., Asghari A., Jamaati-e-Somarin S.H., Saeidi M., Zabihi-e-Mahmoodabad R., Hokmalipour S. (2009). Effects of Water Deficit on Drought Tolerance Indices of Sesame (*Sesamum indicum* L.) Genotypes in Moghan Region. Res. J. Environ. Sci..

[8] Boureima S., Eyletters M., Diouf M., Diop T.A., Van Damme P. (2011). Sensitivity of Seed Germination and Seedling Radicle Growth to Drought Stress in Sesame, *Sesamum indicum* L.. Res. J. Environ. Sci..

[9] Dossa K., Diouf D., Cissé N. (2016). Genome-Wide Investigation of Hsf Genes in Sesame Reveals Their Segmental Duplication Expansion and Their Active Role in Drought Stress Response. Front. Plant Sci..

[10] Dossa K., Yehouessi L., Likeng-Li-Ngue B., Diouf D., Liao B., Zhang X., Cissé N., Bell J. (2017). Comprehensive Screening of Some West and Central African Sesame Genotypes for Drought Resistance Probing by Agromorphological, Physiological, Biochemical and Seed Quality Traits. Agronomy.

[11] Bahrami H., Razmjoo J., Jafari A.O. (2012). Effect of Drought Stress on Germination and Seedling Growth of Sesame Cultivars (*Sesamum indicum* L.). Int. J. AgriScience.

[12] Dissanayake I., Ranwala S.M.W., Perera S.S.N. (2020). Germination and Seedling Growth Responses of Sri Lankan Grown Sesame/Thala (*Sesamum indicum* L.) for Simulated Drought Conditions. J. Natl. Sci. Found. Sri Lanka.

[13] Kermani S.G., Saeidi G., Sabzalian M.R., Gianinetti A. (2019). Drought stress influenced sesamin and sesamolin content and polyphenolic components in sesame (*Sesamum indicum* L.) populations with contrasting seed coat colors. Food Chem..

[14] Mundim F.M., Pringle E.G. (2018). Whole-plant metabolic allocation under water stress. Front. Plant Sci..

[15] Najafabadi M.Y., Ehsanzadeh P. (2017). Photosynthetic and antioxidative upregulation in drought-stressed sesame (*Sesamum indicum* L.) subjected to foliar-applied salicylic acid. Photosynthetica.

[16] Sun J., Rao Y., Yan T., Yan X., Zhou H. (2010). Effects of drought stress on sesame growth and yield characteristics and comprehensive evaluation of drought tolerance. Chin. J. Oil Crop Sci..

[17] Boureima S., Diouf S., Amoukou M., Van Damme P. (2016). Screening for sources of tolerance to drought in sesame induced mutants: Assessment of indirect selection criteria for seed yield. Int. J. Pure Appl. Biosci..

[18] Norsworthy J.K., Oliveira M.J. (2006). Sicklepod (*Senna obtusifolia*) germination and emergence as affected by environmental factors and seeding depth. Weed Sci..

[19] Mensah J.K., Obadoni B.O., Eruotor P.G., Onome-Irieguna F. (2006). Simulated Flooding and Drought Effects on Germination, Growth, and Yield Parameters of Sesame (*Sesamum indicum* L.). Afr. J. Biotechnol..

[20] El Harfi M., Hanine H., Rizki H., Latrache H., Nabloussi A. (2016). Effect of Drought and Salt Stresses on Germination and Early Seedling Growth of Different Color-Seeds of Sesame (*Sesamum indicum*). Int. J. Agric. Biol..

[21] Zraibi L., Nabloussi A., Kajeiou M., Elamrani A., Khalid A., Caid H.S. (2011). Comparative Germination and Seedling Growth Response to Drought and Salt Stresses in a Set of Safflower (*Carthamus tinctorius*) Varieties. Seed Technol..

[22] Office Régional de Mise en Valeur Agricole, ORMVA-Tadla. https://www.ormva-tadla.ma/ormvat/office.

[23] El Harfi M., Jbilou M., Hanine H., Rizki H., Fechtali M., Nabloussi A. (2018). Genetic Diversity Assessment of Moroccan Sesame (*Sesamum indicum* L.) Populations Using Agro-Morphological Traits. J. Agric. Sci. Technol. A.

[24] El Harfi M., Charafi J., Houmanat K., Hanine H., Nabloussi A. (2021). Assessment of Genetic Diversity in Moroccan Sesame (*Sesamum indicum*) Using ISSR Molecular Markers. OCL.

[25] Kouighat M., Channaoui S., Labhilili M., El Fechtali M., Nabloussi A. (2020). Novel Genetic Variability in Sesame Induced via Ethyl Methane Sulfonate. J. Crop. Improv..

[26] Yigit N., Sevik H., Cetin M., Kaya N. (2016). Determination of the effect of drought stress on the seed germination in some plant species. Water Stress Plants.

[27] Ávila M.R., de Braccini A.L., Scapim C.A., Fagliari J.R., dos Santos J.L. (2007). Influência Do Estresse Hídrico Simulado Com Manitol Na Germinação de Sementes e Crescimento de Plântulas de Canola. Rev. Bras. Sementes.

[28] Mohammadi G.R., Amiri F. (2010). The Effect of Priming on Seed Performance of Canola (*Brassica napus* L.) under Drought Stress. Am. Eurasian J. Agric. Environ. Sci..

[29] Channaoui S., El Idrissi I.S., Mazouz H., Nabloussi A. (2019). Reaction of Some Rapeseed (*Brassica napus* L.) Genotypes to Different Drought Stress Levels during Germination and Seedling Growth Stages. OCL.

[30] Luan Z., Xiao M., Zhou D., Zhang H., Tian Y., Wu Y., Guan B., Song Y. (2014). Effects of Salinity, Temperature, and Polyethylene Glycol on the Seed Germination of Sunflower (*Helianthus annuus* L.). Sci. World J..

[31] Botía P., Carvajal M., Cerdá A., Martínez V. (1998). Response of Eight *Cucumis melo* Cultivars to Salinity during Germination and Early Vegetative Growth. Agronomie.

[32] Almas D.E., Bagherikia S., Mashaki K.M. (2013). Effects of Salt and Water Stresses on Germination and Seedling Growth of *Artemisia vulgaris* L.. Int. J. Agric. Crop. Sci..

[33] Gill P.K., Sharma A.D., Singh P., Bhullar S.S. (2003). Changes in Germination, Growth and Soluble Sugar Contents of *Sorghum bicolor* (L.) Moench Seeds under Various Abiotic Stresses. Plant Growth Regul..

[34] McDonald M.B. (2007). Physiology of Seed Germination.

[35] Haouari C.C., Nasraoui A.H., Carrayol E., Gouia H. (2013). Variations In Amylase and-Glycosidase Activities in Two Genotypes of Wheat under NaCl Salinity Stress. Afr. J. Agric. Res..

[36] Ahmad S., Ahmad R., Ashraf M.Y., Ashraf M., Waraich E.A. (2009). Sunflower (*Helianthus annuus* L.) Response to Drought Stress at Germination and Seedling Growth Stages. Pak. J. Bot..

[37] Gordin C.R.B., Scalon S.D.P.Q., Masetto T.E. (2015). Disponibilidade Hídrica Do Substrato e Teor de Água Da Semente Na Germinação de Niger. Pesqui. Agropecu. Trop..

[38] Bakhshandeh E., Jamali M., Afshoon E., Gholamhossieni M. (2017). Using Hydrothermal Time Concept to Describe Sesame (*Sesamum indicum* L.) Seed Germination Response to Temperature and Water Potential. Acta Physiol. Plant.

[39] Santarém E.R., Almeida-Cortez J.S., da Silveira T., Ferreira A.G. (1996). Efeito Do Estresse Hídrico Na Germinação e Crescimento Inicial de Três Espécies de Leguminosas. Acta Bot. Bras..

[40] Okçu G., Kaya M.D., Atak M. (2005). Effects of Salt and Drought Stresses on Germination and Seedling Growth of Pea (P*isum sativum* L.). Turk. J. Agric. For..

[41] Chimungu J.G., Brown K.M., Lynch J.P. (2014). Reduced root cortical cell file number improves drought tolerance in maize. Plant Physiol.

[42] Bor M., Seckin B., Ozgur R., Yılmaz O., Ozdemir F., Turkan I. (2009). Comparative Effects of Drought, Salt, Heavy Metal and Heat Stresses on Gamma-Aminobutryric Acid Levels of Sesame (*Sesamum indicum* L.). Acta Physiol. Plant.

[43] Saxena D., Flores S., Stotzky G. (2002). Bt Toxin is Released in Root Exudates from 12 Transgenic Corn Hybrids Representing Three Transformation Events. Soil Biol. Biochem..

[44] Sikder S., Hasan M.A., Hossain M.S. (2009). Germination Characteristics and Mobilization of Seed Reserves in Maize Varieties as Influenced by Temperature Regimes. J. Agric. Rural. Dev..

[45] Mir R.R., Zaman-Allah M., Sreenivasulu N., Trethowan R., Varshney R.K. (2012). Integrated genomics, physiology and breeding approaches for improving drought tolerance in crops. Theor. Appl. Genet..

[46] Ahmed H.G.M.D., Sajjad M., Li M., Azmat M.A., Rizwan M., Maqsood R.H., Khan S.H. (2019). Selection criteria for drought-tolerant bread wheat genotypes at seedling stage. Sustainability.

[47] Dhanda S.S., Sethi G.S., Behl R.K. (2004). Indices of Drought Tolerance in Wheat Genotypes at Early Stages of Plant Growth. J. Agron. Crop. Sci..

[48] Spielmeyer W., Hyles J., Joaquim P., Azanza F., Bonnett D., Ellis M.E., Moore C., Richards R.A. (2007). A QTL on Chromosome 6A in Bread Wheat (*Triticum aestivum*) is Associated with Longer Coleoptiles, Greater Seedling Vigour and Final Plant Height. Theor. Appl. Genet..

[49] Koskosidis A., Khah E., Mavromatis A., Pavli O., Vlachostergios D.N. (2020). Effect of PEG-Induced Drought Stress on Germination of Ten Chickpea (*Cicer arietinum* L.) Genotypes. Not. Bot. Horti Agrobot. Cluj-Napoca.

[50] Michel B.E., Kaufmann M.R. (1973). The Osmotic Potential of Polyethylene Glycol 6000. Plant Physiol..

[51] ISTA (1999). International Seed Testing Association. Seed Sci. Technol..

[52] Farooq M., Wahid A., Kobayashi N., Fujita D., Basra S.M.A. (2009). Plant Drought Stress: Effects, Mechanisms and Management. Sustainable Agriculture.

[53] Sadeghi H., Khazaei F., Yari L., Sheidaei S. (2011). Effect of seed osmopriming on seed germination behavior and vigor of soybean (*Glycine max* L.). ARPN J. Agric. Biol. Sci..

