# Supplementary material for: First Report of Sesame Mutants Tolerant to Severe Drought Stress during Germination and Early Seedling Growth Stages
Source: Plants (Basel). 2021 Jun 8;10(6):1166. doi: 10.3390/plants10061166 (PMC8227276; doi:10.3390/plants10061166)
Supplement: Supplementary file 1 [file plants-10-01166-s001.zip › plants-1159937-supplementary.pdf]

Table S1: The average values of all parameters, the germination percentage (GP), the germination rate (GR), mean germination time (MGT), root length (RL), shoot length (SL), root-to-shoot ratio (RSR) and seedling vigor index (SVI) of 13 sesame genotypes evaluated under drought stress for two seed generations M_2 and M_3.

		**Germination percentage (GP)**			**Germination rate (GR)**			**Mean germination Time (MGT)**			**Seedling vigor index (SVI)**			**Root length (RL)**			**Shoot length (SL)**			**Root/shoot ratio (RSR)**		
**Genotype**	**Generation**	0 MPa	-0.6 MPa	-1.2 MPa	0 MPa	-0.6 MPa	-1.2 MPa	0 MPa	-0.6 MPa	-1.2 MPa	0 MPa	-0.6 MPa	-1.2 MPa	0 MPa	-0.6 MPa	-1.2 MPa	0 MPa	-0.6 MPa	-1.2 MPa	0 MPa	-0.6 MPa	-1.2 MPa
**US06**	M_2	99.33	69.25	0.00	95.14	63.12	0.00	0.010 5	0.0354	0.000	302. 45	135.44	0.00	3.32	4.9	0.00	4.82	1.27	0.00	0.85	0.90	0.00
	M_3		65.48	0.00		52.48	0.00		0.0334	0.000		136.78	0.00		5.00	0.00		1.17	0.00		1.05	0.00
**US1-3**	M_2	97.00	73.69	15.03	98.47	73.14	23.00	0.010 1	0.0186	0.073	175. 36	122.48	0.00	3.44	5.06	1.06	5.48	1.16	0.00	0.77	1.11	0.00
	M_3		81.25	21.33		70.35	20.55		0.0192	0.078		121.98	0.00		5.33	0.97		1.22	0.00		1.29	0.00
**US1-DL**	M_2	98.00	71.65	18.33	97.82	78.36	16.59	0.011	0.0177	0.090	272. 15	150.81	38.19	4.7	6.21	0.41	4.11	2.14	0.73	1.03	1.2327	1.45
	M_3		85.17	18.85		72.17	17.09		0.0188	0.088		142.46	34.99		5.20	0.39		1.46	0.67		1.02	1.44
**US2-7**	M_2	98.33	58.64	8.66	99.27	48.14	13.33	0.010 0	0.0462	0.105	212. 69	144.89	0.00	3.56	4.31	0.31	5.50	1.56	0.00	0.79	0.39	0.00
	M_3		25.78	0.00		12.78	0.00		0.0367	0.000		146.74	0.00		3.15	0.00		1.41	0.00		0.35	0.00
**US1-2**	M_2	97.33	55.48	0.00	26.24	42.60	0.00	0.010 3	0.0234	0.124	320. 63	140.13	15.03	3.85	5.12	0.00	5.90	1.98	0.41	1.22	0.76	1.00
	M_3		18.74	0.00		25.36	0.00		0.0394	0.000		140.4	17.34		5.30	0.00		1.99	0.53		0.85	0.73
**US2-1**	M_2	99.01	60.34	0.00	98.87	49.36	0.00	0.010 1	0.0202	0.000	252. 66	119.89	0.00	4.32	5.32	0.00	5.36	1.24	0.00	0.95	1.06	0.00
	M_3		58.47	0.00		45.47	0.00		0.0219	0.000		115.76	0.00		5.42	0.00		1.1	0.00		1.10	0.00
**US2-6**	M_2	97.33	52.17	0.00	92.15	33.45	0.00	0.010 8	0.0480	0.000	293. 24	123.22	5.55	3.35	4.84	0.00	5.47	1.43	0.33	0.75	0.78	0.93
	M_3		27.35	0.00		14.35	0.00		0.0560	0.000		114.96	0.00		3.78	0.00		1.1	0.00		0.90	0.00
**ML13**	M_2	97.02	67.99	0.00	98.70	67.48	0.00	0.010 1	0.0297	0.000	242. 87	162.47	0.00	3.92	5.66	0.00	4.69	1.36	0.00	0.81	0.78	0.00
	M_3		73.05	0.00		56.14	0.00		0.0320	0.000		166.85	0.00		5.74	0.00		1.42	0.00		0.82	0.00
**ML2-10**	M_2	100.0 0	80.36	24.00	98.97	72.14	21.9	0.010 1	0.0138	0.075	238. 94	173.25	9.10	4.34	6.33	2.33	4.98	1.83	0.53	1.03	1.47	0.47
	M_3		85.69	37.91		78.64	24.2		0.0127	0.071		175.14	12.67		6.25	2.05		1.71	0.64		1.39	0.51
**ML2-5**	M_2	97.66	85.78	38.33	97.58	82.30	41.08	0.010 2	0.0121	0.054	262. 01	205.64	92.40	4.52	6.35	2.35	4.60	2.2	1.52	1.15	1.49	1.53
	M_3		83.55	35.02		80.25	39.93		0.0124	0.055		192.93	121.69		6.11	2.11		2.34	1.16		1.54	1.76
**ML2-37**	M_2	97.96	1.32	7.33	92.95	68.99	21.06	0.010 6	0.0203	0.077	260. 26	225.48	145.67	3.99	5.36	1.05	5.30	1.87	1.45	0.90	1.26	1.62
	M_3		77.17	9.05		64.17	26.13		0.0189	0.063		243.2	116.97		5.63	0.94		2.4	1.23		1.32	1.71
**ML2-72**	M_2	98.66	57.26	11.67	97.88	45.69	15.67	0.010 2	0.0218	0.093	241. 85	127.87	8.58	3.86	4.64	0.25	5.22	1.53	0.63	0.89	0.61	1.02
	M_3		61.25	13.06		48.25	12.15		0.0207	0.011		135.45	10.50		3.42	0.33		1.68	0.74		0.55	1.12
**ML2-68**	M_2	96.96	42.6	0.00	96.78	33.69	0.00	0.010 3	0.0296	0.000	257. 25	122.94	0.00	3.77	4.72	0.00	4.95	1.45	0.00	0.92	0.69	0.00
	M_3		29.78	0.00		34.00	0.00		0.0294	0.000		128.65	0.00		4.98	0.00		1.31	0.00		0.94	0.00
